# A comparison of menotropin, highly-purified menotropin and follitropin alfa in cycles of intracytoplasmic sperm injection

**DOI:** 10.1186/1477-7827-7-111

**Published:** 2009-10-14

**Authors:** Sandro C Esteves, Joan C Schertz, Sidney Verza, Danielle T Schneider, Silval FC Zabaglia

**Affiliations:** 1 ANDROFERT - Centro de Referência para Reprodução Masculina, Av. Dr. Heitor Penteado, 1464, 13075-460 Campinas, São Paulo, Brazil; 2 EMD Serono, Rockland (an affiliate of Merck KGaA, Darmstadt, Germany), MA, USA

## Abstract

**Background:**

Over the last several decades, as a result of an evolution in manufacturing processes, a marked development has been made in the field of gonadotropins for ovarian stimulation. Initially, therapeutic gonadotropins were produced from a simple process of urine extraction and purification; now they are produced via a complex system involving recombinant technology, which yields gonadotropins with high levels of purity, quality, and consistency.

**Methods:**

A retrospective analysis of 865 consecutive intracytoplasmic sperm injection (ICSI) cycles of controlled ovarian hyperstimulation (COH) compared the clinical efficacy of three gonadotropins (menotropin [hMG; n = 299], highly-purified hMG [HP-hMG; n = 330] and follitropin alfa [r-hFSH; n = 236]) for ovarian stimulation after pituitary down-regulation. The endpoints were live birth rates and total doses of gonadotropin per cycle and per pregnancy.

**Results:**

Laboratory and clinical protocols remained unchanged over time, except for the type of gonadotropin used, which was introduced sequentially (hMG, then HP-hMG, and finally r-hFSH). Live birth rates were not significantly different for hMG (24.4%), HP-hMG (32.4%) and r-hFSH (30.1%; p = 0.09) groups. Total dose of gonadotropin per cycle was significantly higher in the hMG (2685 +/- 720 IU) and HP-hMG (2903 +/- 867 IU) groups compared with the r-hFSH-group (2268 +/- 747 IU; p < 0.001). Total dose of gonadotropin required to achieve clinical pregnancy was 15.7% and 11.0% higher for the hMG and HP-hMG groups, respectively, compared with the r-hFSH group, and for live births, the differences observed were 45.3% and 19.8%, respectively.

**Conclusion:**

Although similar live birth rates were achieved, markedly lower doses of r-hFSH were required compared with hMG or HP-hMG.

## Background

Over the last several decades, a marked development has been made in the field of gonadotropins for ovarian stimulation. This development is the result of an evolution in manufacturing, with the production of therapeutic gonadotropins changing from a simple process of urine extraction and purification to a complex system involving recombinant technology, yielding high levels of purity, quality, and consistency. Menotropin (human menopausal gonadotropin [hMG]), labeled as a 1:1 ratio of follicle-stimulating hormone (FSH) and luteinizing hormone (LH) activity, has been widely used since the 1960s. As the purification processes became more sophisticated, other purified gonadotropin products were introduced, including a menotropin formulation (highly-purified hMG; HP-hMG) with the same labeled ratio of FSH:LH activity. In the 1980s, biotechnology advances resulted in the formulation of a recombinant human FSH (r-hFSH), follitropin alfa, the first r-hFSH to become available in the market. Another r-hFSH product, follitropin beta, was also launched [1]. Due primarily to its safety, efficacy, and ease-of-use, r-hFSH soon became the preferred gonadotropin worldwide for controlled ovarian hyperstimulation (COH) in the context of *in vitro *fertilization (IVF) cycles.

In current practice, factors such as efficacy, safety, cost of the medication, ease of administration, and availability of product are the determinants of gonadotropin choice. However, in Brazil and other Latin American countries, assisted reproductive techniques (ART) and the drugs used for ovarian stimulation are neither provided by healthcare plans nor reimbursed by the government. Thus, patients prefer to purchase lower-cost drugs. In recent years, our choice of gonadotropin product was motivated by patient costs and, therefore, we justified the choice of urinary menotropins for COH in ART procedures. When HP-hMG became available, we recommended this product to our patients. However, in addition to safety, efficacy, and purity, follitropin alfa, an r-hFSH product, has a highly consistent isoform profile and low batch-to-batch variability that may be associated with a more predictable follicular development [2]. Therefore, in 2004, we selected r-hFSH as the drug of choice for COH in intracytoplasmic sperm injection (ICSI) cycles.

The objective of the current analysis was to assess treatment outcomes of ART procedures at our center to compare the efficacy of hMG, HP-hMG, and r-hFSH (follitropin alfa filled-by-mass [FbM]) for COH after pituitary down-regulation in ICSI cycles.

## Methods

Consecutive ICSI cycles (n = 865) performed at Androfert, a tertiary referral center for male infertility treatment in Campinas, Brazil, from April 2000 to November 2005 were included in this analysis. Laboratory and clinical protocols remained unchanged during these time periods, except for the type of gonadotropin that was sequentially changed over time, as follows: hMG (Menogon^®^, Ferring, Brazil) from April 2000 to February 2002; HP-hMG (Menopur^®^, Ferring, Brazil) from March 2002 to January 2004; and r-hFSH (GONAL-f^® ^FbM, Merck Serono, Brazil) from February 2004 to present. During the considered period, COH was performed in 299 cycles with hMG; in 330 cycles with HP-hMG; and in 236 cycles with r-hFSH.

Patient cycle data were entered into a database using customized software (Androsys^®^, Androfert-UNIOESTE, Brazil). The overall outcomes of our IVF program have been consistent during the study period (Table 1).

**Table 1:** Main outcome measures for ANDROFERT, São Paulo reported to REDLARA, 2002-2005* (ICSI cycles only).

	**2002**	**2003**	**2004**	**2005**
Number of cycles^†^	167	164	151	195
Mean number of transferred embryos per patient	3.3	3.2	3.4	3.3
Clinical pregnancy rate per transfer, %	37.0	46.8	46.7	40.3
Ectopic pregnancy, %	0.7	0.7	2.1	2.6
Spontaneous abortion, %	26.7	19.7	20.6	13.6
Live birth rate, %	28.5	39.7	30.0	30.1
Indication for ICSI, %				
Male	26.8	28.2	35.2	32.5
Female	23.0	9.4	24.8	17.2
Both	50.2	62.4	40.0	50.3

*Center began reporting to REDLARA in 2002.

^† ^Total number of ICSI cycles reported is 677 - the cycles prior to 2002, when reporting began, are not included in this table.

ICSI, intracytoplasmic sperm injection; REDLARA, Latin American Registry.

Signed informed consent was obtained from patients to use both clinical and laboratory data for analysis. The analysis was approved by our Institutional Review Board. Indications for ICSI were in accordance with the guidelines of the II Brazilian Consensus of Male Infertility [3], even if the indication for IVF was a female factor.

### Ovarian stimulation

The protocol of ovarian stimulation was the same for all cycles, except for the gonadotropin product. For all cycles, treatment was initiated with 400 μg daily intranasal administration of gonadotropin-releasing hormone agonist (GnRH-a; nafarelin acetate, Synarel^®^, Zodiac, Brazil), starting on the twenty-first day of the menstrual cycle and was maintained until the day before human chorionic gonadotropin (hCG) administration. For ovarian stimulation, initial daily doses of 150-375 IU of hMG, HP-hMG or r-hFSH were used and no patients were treated with combinations of hMG or HP-hMG plus r-hFSH. The initial dose of gonadotropin was determined by the treating physician taking into account: age, body mass index, serum FSH on day 2 or 3 of the menstrual cycle, baseline ovarian volume on transvaginal ultrasound (TVUS), and number of pre-antral follicles between days 2 and 3 of the menstrual cycle after pituitary down-regulation. Ovarian stimulation commenced only after confirming pituitary down-regulation by both serum estradiol levels <50 pg/mL and the absence of ovarian follicles >10 mm in diameter on TVUS.

Ultrasound assessment between the sixth and eighth days of stimulation was performed to determine if gonadotropin dose adjustments were required; if prevention of ovarian hyper-response was deemed necessary, the dose was reduced. There was no dose increase of gonadotropin during stimulation, even in cases of poor ovarian response. hCG was administered when two or more ovarian follicles reached a mean diameter of 18 mm. In the cycles with hMG and HP-hMG, urine-derived hCG 10,000 IU was used (Choragon^®^, Ferring, Brazil), while recombinant choriogonadotropin 250 μg (Ovidrel^® ^lyophilized, Merck Serono, Brazil) was used following stimulation with r-hFSH.

### Oocyte retrieval, sperm processing, and IVF

Oocyte retrieval, sperm processing, and IVF were carried out as previously reported [4-6]. Briefly, oocyte retrieval was performed under intravenous sedation with propofol and guided by TVUS, 34-36 hours after hCG administration. After oocyte aspiration, follicular fluid was examined for cumulus-corona-oocyte complexes. The complexes were chemically denuded with 40 IU/mL of hyaluronidase (Hyase^®^, Vitrolife, Sweden). The isolated oocytes were then mechanically denuded and classified according to nuclear maturity. The oocytes were maintained in culture until sperm microinjection [4].

Spermatozoa obtained by ejaculation were collected after a 48-72 hour period of ejaculatory abstinence. Sperm processing was performed by the two-layer discontinuous colloidal gradient [5]. In the cases of azoospermia, either testicular sperm aspiration, percutaneous epididymal sperm aspiration, or testicular sperm extraction using microsurgery were performed for sperm retrieval [6]. Selection and immobilization of the spermatozoon, and microinjections, were performed under a 400× magnification [4]. The injected oocytes were transferred to a closed culture system and incubated for 16-18 hours at 37°C and 5.5% CO_2_, until confirmation of fertilization. Fertilization was considered normal when oocytes with two pronuclei were seen.

### Embryo culture, transfer, and cleavage check

Fertilized oocytes were maintained in culture until transfer of the embryos to the uterine cavity guided by abdominal ultrasound on the third day of embryo culture. The embryos were placed 10-15 mm from the fundus of the uterine cavity.

Embryo cleavage was checked approximately 48 and 72 hours after ICSI and the number, symmetry, and expansion of the blastomeres, multinucleation, anomalies of the zona pellucida, and the rate of cytoplasmic fragmentation were recorded. The embryos were classified as top quality when they had three to four symmetrical blastomeres on the second day of culture and seven to eight symmetrical blastomeres on the third day, with no multinucleation, grade I (no fragmentation) or grade II fragmentation (up to 20% of the perivitelline space with fragments), and no abnormalities in the zona pellucida [7].

Oocyte collection, micromanipulation of gametes, embryo culture, and the transfer of embryos to the uterine cavity were carried out under ISO 6, ISO 5, and ISO 7 environments, respectively (VECO, Brazil) [8].

### Statistical analysis

All ICSI cycles in our database, within the specified dates, were included in the analysis, with no inclusion or exclusion criteria. For the analysis of the main outcomes (live birth rates, and the total doses of gonadotropin per cycle, per clinical pregnancy, and per live birth), the data were stratified to the prognostic factors commonly used in reproductive medicine (age, infertility factor, and number of prior cycles).

The total population was initially analyzed descriptively. Besides the main outcomes and the criteria used for stratification, the following outcomes were also evaluated: duration of stimulation; cancellation rates due to poor ovarian response (defined as <3 follicles ≥10 mm and/or a serum estradiol level of <100 pg/mL on stimulation day 7 via ultrasound scan); baseline serum FSH and LH levels (obtained on days 2 or 3 of the previous menstrual cycle); serum estradiol levels on the day of hCG administration; incidence of serious ovarian hyperstimulation syndrome (OHSS) as per the Golan criteria [9]; number of aspirated oocytes; number of mature oocytes; rate of fertilization; percent of top quality embryos (per total number of embryos obtained); number of transferred embryos; implantation rates (number of fetal sacs per embryos transferred); pregnancy rates; and miscarriage rates. The main outcomes were analyzed both as a whole and standardized for age, infertility rate, and number of prior ICSI cycles.

The qualitative variables were expressed as both absolute (n) and relative (%) frequencies; the quantitative variables were means and standard deviations. The Kolmogorov-Smirnov test was applied to check the normal distribution through numeric variables. The relationship among the variables was evaluated by the Chi-square test. The analysis of variance for one factor (one-way ANOVA) was used for the comparison of quantitative variables when there was a normal distribution of each variable in the gonadotropin treatment groups. Differences were analyzed by the Tukey multiple comparisons test. For the variables without normal distribution, comparisons were performed by the Kruskal-Wallis test and the differences were compared using the Dunn multiple comparisons test. A p value of below 0.05 was considered significant.

For each group, the mean total dose of gonadotropin administered per cycle was divided by both the clinical pregnancy and live birth rates, in order to define the total dose of gonadotropin necessary to achieve one pregnancy or one live birth. Multiple pregnancies were considered as a single event.

When one gestation produced one live birth and one abortion, the result was registered as a live birth. It was not possible to calculate the statistical significance for the necessary doses for achieving pregnancies and live births among the groups, due to methodological reasons. However, the differences regarding the gonadotropin doses used in the subgroups with pregnancies and live births could be statistically compared among the groups.

## Results

The distribution of the 865 ICSI cycles (age, indication, baseline serum FSH and LH levels, and total number of ICSI cycles) is presented in Table 2. The mean ± standard deviation (SD) patient age was significantly lower in the hMG group (32.5 ± 5.3 years) compared with patients in the HP-hMG and r-hFSH groups (34.0 ± 4.7 and 34.3 ± 4.8; p < 0.001, respectively). Even after stratification by patient age groups, 71.2% of patients were ≤35 years old in the hMG group (mean ± SD age = 29.9 ± 3.7 years), compared with 60.0% (mean ± SD age = 30.9 ± 3.2 years) and 60.2% (mean ± SD age = 30.9 ± 3.2 years; p = 0.005) of the HP-hMG and r-hFSH groups, respectively. In the >35 years old patient subgroup, the mean ± SD baseline LH levels were significantly lower in the hMG and r-hFSH groups (4.2 ± 3.0 and 4.3 ± 3.9, respectively) than in the HP-hMG group (5.8 ± 4.8; p = 0.004). Baseline FSH was not significantly different between groups.

**Table 2:** Baseline details of the 865 ICSI cycles.

	**hMG** **(n = 299)**	**HP-hMG** **(n = 330)**	**r-hFSH** **(n = 236)**	**p value**
Mean age, years (± SD)	32.5 (± 5.3)^a^	34.0 (± 4.7)^b^	34.3 (± 4.8)^c^	< 0.001 (^a ^vs ^b, c^)
≤35 years, n (%)	213 (71.2)^a^	198 (60.0)^b^	142 (60.2)^c^	0.005 (^a ^vs ^b, c^)
>35 years, n (%)	86 (28.8)^a^	132 (40.0)^b^	94 (39.8)^c^	0.005 (^a ^vs ^b, c^)
Indication, n (%)				
Male factor only	111 (37.1)	99 (30.0)	76 (32.2)	0.27
Mixed factor*	142 (47.5)	156 (47.3)	129 (54.7)	0.19
Female factor	46 (15.4)	75 (22.7)	31 (13.1)	0.33
Treatment cycle, n (%)				
1st cycle	195 (65.3)	192 (58.1)	145 (61.4)	0.49
2nd cycle	80 (26.7)	86 (26.0)	60 (25.4)	0.83
≥3rd cycle	24 (8.0)	52 (15.9)	31 (13.2)	0.34
Mean baseline FSH, IU/L (± SD)	6.3 (± 3.2)	6.7 (± 3.7)	6.5 (± 2.8)	0.33
≤35 years	6.0 (± 2.8)	6.5 (± 3.1)	6.1 (± 3.0)	0.23
>35 years	7.2 (± 3.9)	7.2 (± 4.4)	7.3 (± 4.7)	0.69
Mean baseline LH, IU/L (± SD)	4.9 (± 4.1)	5.4 (± 4.4)	5.2 (± 3.7)	0.23
≤35 years	5.2 (± 4.4)	5.2 (± 4.1)	5.1 (± 3.9)	0.63
>35 years	4.2 (± 3.0)^a^	5.8 (± 4.8)^b^	4.3 (± 3.9)^c^	0.004 (^b ^vs ^a, c^)

Distribution by menotropin (hMG), highly-purified menotropin (HP-hMG) or follitropin alfa (r-hFSH), after pituitary down-regulation, in relation to the age, indication, baseline FSH and LH levels, and total number of treated cycles.

*Mixed factor: male + female.

FSH, follicle stimulating hormone; hMG, human menopausal gonadotropin; HP-hMG, highly-purified hMG; ICSI, intracytoplasmic sperm injection; LH, luteinizing hormone; r-hFSH, recombinant human FSH; SD, standard deviation.

The clinical and laboratory outcomes are provided in detail in Table 3. The length of stimulation was significantly shorter in the hMG group in comparison with the other groups, both for the total group and the age-stratified subgroups. On the day of hCG administration, the serum estradiol levels were significantly different between the different gonadotropin product groups and for the age-stratified subgroups as well. On the other hand, the number of metaphase II oocytes in patients >35 years of age treated with hMG was significantly lower when compared with the other treatment groups. The percentage of top quality embryos available for transfer was significantly higher in the HP-hMG group, both for the total group and the subgroup ≤35 years of age. The cancellation rates due to poor ovarian response and the incidence of serious OHSS did not differ among the groups.

**Table 3:** Clinical and laboratory outcomes of COH during the 865 ICSI cycles.

	**hMG** **(n = 299)**	**HP-hMG** **(n = 330)**	**r-hFSH** **(n = 236)**	**p value**
Mean length of stimulation, days (± SD)	9.6 (± 1.3)^a^	10.0 (± 1.3)^b^	10.1 (± 1.0)^c^	< 0.001 (^a ^vs ^b, c^)
≤35 years	9.5 (± 1.3)^a^	9.9 (± 1.2)^b^	10.0 (± 0.9)^c^	< 0.001 (^a ^vs ^b, c^)
>35 years	9.8 (± 1.4)^a^	10.2 (± 1.6)^b^	10.2 (± 1.1)^c^	0.007 (^a ^vs ^b, c^)
Gonadotropin dose adjustment, n (%)	56 (18.7)^a^	67 (20.3)^b^	126 (53.4)^c^	< 0.001 (^c ^vs ^a, b^)
≤35 years	43 (20.1)^a^	47 (23.7)^b^	90 (63.3)^c^	< 0.001 (^c ^vs ^a, b^)
>35 years	13 (15.1)^a^	20 (15.1)^b^	36 (38.2)^c^	< 0.001 (^c ^vs ^a, b^)
Mean estradiol on day of hCG, pg/mL (± SD)	1967.0 (± 1241.0)^a^	2479.0 (± 1708.0)^b^	2159.3 (± 1411.7)^c^	0.001 (^a ^vs ^b, c^)
≤35 years	2174.8 (± 1224.4)^a^	2609.3 (± 1632.0)^b^	2392.3 (± 1422.3)^c^	0.035 (^a ^vs ^b^)
>35 years	1432.7 (± 1124.6)^a^	2285.1 (± 1804.4)^b^	1789.1 (± 1325.7)^c^	0.001 (^a ^vs ^b^)
Cancellation rate, n (%)	23 (7.7)	21 (6.4)	17 (7.2)	0.80
≤35 years	10 (4.7)	9 (4.5)	10 (7.0)	0.50
>35 years	13 (15.1)	12 (9.0)	7 (7.4)	0.20
Severe OHSS, n (%)	7 (2.3)	6 (1.8)	3 (1.3)	0.76
≤35 years, n	5	4	3	-
>35 years, n	2	2	0	-
Mean number of retrieved oocytes, n (± SD)	10.9 (± 6.8)	10.7 (± 6.5)	10.8 (± 6.7)	0.97
≤35 years	12.3 (± 6.7)	11.3 (± 5.5)	11.7 (± 6.0)	0.50
>35 years	7.5 (± 5.8)	9.7 (± 7.6)	9.5 (± 7.5)	0.08
Mean MII oocytes, n (± SD)	8.9 (± 5.6)	8.9 (± 5.7)	8.7 (± 5.6)	0.82
≤35 years	10.1 (± 5.5)	9.4 (± 4.8)	9.1 (± 5.0)	0.20
>35 years	5.8 (± 4.4)^a^	8.3 (± 6.8)^b^	8.1 (± 6.3)^c^	0.01 (^a ^vs ^b, c^)
Fertilization rate 2PN, % (± SD)	72 (± 25)	72 (± 22)	71 (± 23)	0.83
≤35 years	71 (± 22)	72 (± 22)	72 (± 22)	0.76
>35 years	73 (± 30)	72 (± 24)	70 (± 25)	0.20
Top quality embryos on day3, % (± SD)	40 (± 30)^a^	47 (± 31)^b^	39 (± 29)^c^	0.004 (^b ^vs ^a, c^)
≤35 years	41 (± 29)^a^	47 (± 28)^b^	39 (± 28)^c^	0.03 (^b ^vs ^a, c^)
>35 years	39 (± 34)	48 (± 34)	37 (± 30)	0.08
Mean number of transferred embryos, n (± SD)	3.4 (± 1.6)	3.4 (± 1.5)	3.2 (± 1.6)	0.18
≤35 years	3.5 (± 1.4)	3.5 (± 1.4)	3.2 (± 1.4)	0.05
>35 years	2.9 (± 1.9)	3.1 (± 1.6)	3.2 (± 1.8)	0.50

Distribution by menotropin (hMG), highly-purified menotropin (HP-hMG) or follitropin alfa (r-hFSH) for COH. Overall and age-stratified outcomes are presented.

2PN, two pronuclei; COH, controlled ovarian hyperstimulation; human chorionic gonadotropin; hMG, human menopausal gonadotropin; HP-hMG, highly-purified hMG; ICSI, intracytoplasmic sperm injection; MII, metaphase II; OHSS, ovarian hyperstimulation syndrome; r-hFSH, recombinant human follicle-stimulating hormone; SD, standard deviation.

The clinical pregnancy rates per initiated cycle, implantation and live birth rates per initiated cycle were not statistically different among the groups (Table 4). However, the differences seen in the live birth rates were in favor of the r-hFSH and HP-hMG groups, for both the total population (p = 0.09) and the subgroup of patients ≤35 years of age (p = 0.08; Table 4), although these differences were not statistically significant. After stratification for indication and number of ICSI attempts, there was no difference in the live birth rates among the groups (data not shown, p = 0.16). The incidence of spontaneous abortion was significantly higher in the hMG group compared with the other groups (p = 0.009), mainly due to the subgroup of patients >35 years of age (Table 4).

**Table 4:** Pregnancy outcomes of the 865 ICSI cycles.

	**hMG** **(n = 299)**	**HP-hMG** **(n = 330)**	**r-hFSH** **(n = 236)**	**p value**
Clinical pregnancy per initiated cycle, n (%)	106 (35.5)	132 (40.0)	82 (34.7)	0.35
≤35 years	84 (39.4)	94 (47.5)	59 (41.5)	0.24
>35 years	22 (25.6)	38 (28.8)	23 (24.5)	0.74
Implantation rate, % (± SD)	16 (24)	20 (27)	16 (23)	0.18
≤35 years	17 (25)	24 (29)	20 (25)	0.15
>35 years	11 (21)	12 (21)	9 (17)	0.61
Ectopic pregnancy, n (%)	1 (0.3)	6 (1.8)	2 (0.8)	-
≤35 years	1	3	1	-
>35 years	0	3	1	-
Spontaneous abortion,* n (%)	33 (31.1)^a^	25 (18.9)^b^	11 (13.4)^c^	0.009 (^a ^vs ^b, c^)
≤35 years	21 (25.0)	16 (17.0)	7 (11.9)	0.12
>35 years	12 (54.5)^a^	9 (23.6)^b^	4 (17.3)^c^	0.01 (^a ^vs ^b, c^)
Live birth per initiated cycle, n(%)	73 (24.4)	107 (32.4)	71 (30.1)	0.09
≤35 years	63 (29.5)	78 (39.4)	52 (36.6)	0.08
>35 years	10 (11.6)	29 (21.9)	19 (20.2)	0.21

Clinical pregnancy per initiated cycle, implantation, ectopic pregnancy and spontaneous abortion rates, and live birth per initiated cycle in 865 ICSI cycles with menotropin (hMG), highly-purified menotropin (HP-hMG) or follitropin alfa (r-hFSH) used for COH. Overall and age-stratified outcomes are presented.

*Spontaneous abortion rates are per clinical pregnancies.

COH, controlled ovarian hyperstimulation; hMG, human menopausal gonadotropin; HP-hMG, highly-purified hMG; ICSI, intracytoplasmic sperm injection; r-hFSH, recombinant human follicle-stimulating hormone; SD, standard deviation.

The clinical and laboratory outcomes of cycles with clinical pregnancy are provided in Table 5. The mean ± SD patient age and the length of stimulation were significantly lower in the hMG group compared with the other groups, both for the total group (p = 0.01) and for the age-stratified subgroup ≤35 years (p = 0.004 and p = 0.001, respectively). On the day of hCG administration, serum estradiol was significantly different between the hMG and HP-hMG groups (p = 0.005) and for all gonadotropin products in the ≤35 years subgroup as well (p = 0.01). The percentage of top quality embryos available for transfer on day 3 was significantly higher in the HP-hMG group, both for the total group (p = 0.004) and the ≤35 years subgroup (p = 0.02). Baseline FSH and LH levels, mean number of retrieved and mature oocytes, fertilization rate, and the mean number of transferred embryos were not significantly different between groups.

**Table 5:** Clinical and laboratory outcomes of COH in ICSI cycles with clinical pregnancy during the 865 cycles.

	**hMG** **(n = 106)**	**HP-hMG** **(n = 132)**	**r-hFSH** **(n = 82)**	**p value**
Mean age, years (± SD)	31.1 (± 5.0)^a^	32.7 (± 4.2)^b^	32.8 (± 4.2)^c^	0.01 (^a ^vs ^b, c^)
≤35 years (± SD)	29.2 (± 3.6)^a^	30.7 (± 3.1)^b^	30.8 (± 2.9)^c^	0.004 (^a ^vs ^b, c^)
>35 years (± SD)	38.3 (± 1.9)^a^	37.0 (± 2.0)^b^	37.3 (± 1.6)^c^	0.04 (^a ^vs ^b, c^)
Mean length of stimulation, days (± SD)	9.6 (± 1.3)^a^	9.8 (± 1.0)^b^	10.0 (± 1.0)^c^	0.01 (^a ^vs ^b, c^)
≤35 years	9.5 (± 1.3)^a^	9.9 (± 1.0)^b^	10.0 (± 0.9)^c^	0.001 (^a ^vs ^b, c^)
>35 years	10.1 (± 1.3)^a^	9.7 (± 1.0)^b^	9.9 (± 1.0)^c^	0.66
Mean baseline FSH, IU/L (± SD)	6.0 (± 2.4)	6.5 (± 3.7)	6.3 (± 2.2)	0.63
≤35 years	5.8 (± 2.2)	6.2 (± 2.9)	6.0 (± 2.0)	0.51
>35 years	6.6 (± 3.2)	7.1 (± 5.0)	7.1 (± 4.5)	0.91
Mean baseline LH, IU/L (± SD)	5.6 (± 5.3)	5.5 (± 3.7)	5.2 (± 3.0)	0.90
≤35 years	5.7 (± 5.6)	5.5 (± 4.0)	5.3 (± 4.1)	0.89
>35 years	5.2 (± 3.5)	5.4 (± 2.7)	5.5 (± 3.1)	0.70
Mean estradiol on day of hCG, pg/mL (± SD)	2102.8 (± 1207.4)^a^	2682.9 (± 1440.7)^b^	2488.0 (± 1630.7)^c^	0.005 (^a ^vs ^b^)
≤35 years	2225.5 (± 1221.0)^a^	2753.3 (± 1402.3)^b^	2742.9 (± 1716.8)^c^	0.01 (^a ^vs ^b, c^)
>35 years	1605.7 (± 1036.6)	2510.7 (± 1537.5)	1850.9 (± 1203.6)	0.06
Mean number of retrieved oocytes, n (± SD)	12.1 (± 6.6)	12.2 (± 6.1)	12.4 (± 6.8)	0.92
≤35 years	13.0 (± 6.7)	12.4 (± 5.2)	12.3 (± 5.9)	0.92
>35 years	8.7 (± 5.1)	11.5 (± 8.0)	12.5 (± 9.0)	0.25
Mean MII oocytes, n (± SD)	9.7 (± 4.8)	10.3 (± 5.5)	9.9 (± 5.5)	0.67
≤35 years	10.6 (± 4.8)	10.3 (± 4.6)	9.7 (± 4.6)	0.20
>35 years	6.4 (± 3.1)	10.1 (± 7.3)	10.6 (± 7.3)	0.05
Fertilization rate 2PN, % (± SD)	77 (± 17)	76 (± 17)	75 (± 18)	0.71
≤35 years	74 (± 17)	74 (± 17)	75 (± 17)	0.91
>35 years	88 (± 12)	79 (± 18)	76 (± 19)	0.07
Top quality embryos on day3, % (± SD)	47 (± 28)^a^	55 (± 28)^b^	41 (± 25)^c^	0.004 (^b ^vs ^a, c^)
≤35 years	49 (± 26)^a^	54(± 28)^b^	40 (± 26)^c^	0.02 (^b ^vs ^c^)
>35 years	40 (± 32)^a^	60 (± 29)^b^	44 (± 22)^c^	0.02 (^b ^vs ^a, c^)
Mean number of transferred embryos, n (± SD)	3.9 (± 1.0)	3.8 (± 0.9)	3.6 (± 0.9)	0.65
≤35 years	3.4 (± 1.0)	3.6 (± 0.9)	3.5 (± 0.9)	0.79
>35 years	4.0 (± 0.9)	4.0 (± 0.8)	3.9 (± 0.9)	0.33

Distribution by menotropin (hMG), highly-purified menotropin (HP-hMG) or follitropin alfa (r-hFSH) for COH. Overall and age-stratified outcomes are presented.

2PN, two pronuclei; COH, controlled ovarian hyperstimulation; FSH, follicle-stimulating hormone; hCG, human chorionic gonadotropin; hMG, human menopausal gonadotropin; HP-hMG, highly-purified hMG; ICSI, intracytoplasmic sperm injection; LH, luteinizing hormone; MII, metaphase II; r-hFSH, recombinant human FSH; SD, standard deviation.

The distribution of live birth cycles in relation to parity, gestational age, and birth weight are shown in Table 6. Singleton births were seen in 68.5%, 63.5%, and 64.8% of live birth cycles in the hMG, HP-hMG and r-hFSH groups, respectively. Significant differences were observed for the gestational age, in the three parity stratifications, in favor of the r-hFSH group compared with the hMG group. In the case of twins and triplets, the difference was significant versus the r-hFSH group (p = 0.03). For triplets, the birth weight was significantly higher in the r-hFSH group versus the hMG group (p = 0.03).

**Table 6:** Live birth outcomes of the 865 ICSI cycles.

	**hMG**	**HP-hMG**	**r-hFSH**	**p value**
Cycles with live births, n	73	107	71	
Single, n (%)	50 (68.5)	68 (63.5)	46 (64.8)	0.57
Twins, n (%)	18 (24.7)	34 (31.8)	22 (31.0)	0.59
Triplets, n (%)	5 (6.8)	5 (4.7)	3 (4.2)	0.89
Mean gestational age, weeks (± SD)				
Single	36.5 (± 3.6)^a^	38.1 (± 2.1)^b^	38.3 (± 2.3)^c^	0.01 (^a ^vs ^b, c^)
Twins	35.0 (± 0.8)^a^	35.1 (± 3.0)^b^	37.3 (± 2.7)^c^	0.03 (^a, b ^vs ^c^)
Triplets	29.3 (± 3.5)^a^	31.1 (± 3.4)^b^	34.0 (± 0.0)^c^	0.03 (^a ^vs ^c^)
Mean weight, g (± SD)				
Single	2882 (± 703)	3064 (± 470)	3024 (± 334)	0.09
Twins	2352 (± 436)^a^	2067 (± 614)^b^	2230 (± 715)^c^	0.01 (^a ^vs ^b^)
Triplets	1250 (± 329)^a^	1470 (± 448)^b^	1747 (± 196)^c^	0.03 (^a ^vs ^c^)

Cycles with live births in relation to the parity and gestational outcomes of the babies from 865 ICSI cycles with menotropin (hMG), highly-purified menotropin (HP-hMG) or follitropin alfa (r-hFSH) used for COH. Overall and parity-stratified outcomes are presented.

COH, controlled ovarian hyperstimulation; hMG, human menopausal gonadotropin; HP-hMG, highly-purified hMG; ICSI, intracytoplasmic sperm injection; r-hFSH, recombinant human follicle-stimulating hormone; SD, standard deviation.

The gonadotropin dose used per cycle was significantly lower in the r-hFSH group compared with the other groups, for both the overall population (p < 0.001) and the stratified subgroups (p < 0.001 and p = 0.02 for the ≤35 years and >35 years subgroups, respectively) (Table 7). These differences were also significant for r-hFSH after stratifying for the cause of infertility (male and mixed factors) and for the number of treatment cycles. Taking into account only the cycles that resulted in clinical pregnancy and live birth, the mean total doses of gonadotropins used for ovarian stimulation were significantly lower for the r-hFSH group in comparison with the hMG and HP-hMG groups.

**Table 7:** Total gonadotropin dose requirements of the 865 ICSI cycles.

	**hMG** **(n = 299)**	**HP-hMG** **(n = 330)**	**r-hFSH** **(n = 236)**	**p value**
Total dose, IU	2685 (± 720)^a^	2903 (± 867)^b^	2268 (± 747)^c^	< 0.001 (^a, b ^vs ^c^)
Age:				
≤35 years	2558 (± 679)^a^	2678 (± 739)^b^	2062 (± 681)^c^	<0.001 (^a, b ^vs ^c^)
>35 years	3002 (± 725)^a^	3240 (± 935)^b^	2783 (± 738)^c^	0.02 (^a, b ^vs ^c^)
Indication:				
Male factor	2582 ± 641^a^	2777 ± 859^b^	2305 ± 606^c^	0.003 (^a, b ^vs ^c^)
Mixed factor	2710 ± 751^a^	2911 ± 892^b^	2439 ± 770^c^	0.02 (^a, b ^vs ^c^)
Number of cycles:				
1	2690 ± 640^a^	2816 ± 791^b^	2374 ± 647^c^	0.003 (^a, b ^vs ^c^)
2	2670 ± 699^a^	2942 ± 1,025^b^	2492 ± 856^c^	0.003 (^a, b ^vs ^c^)
≥3	2601 ± 803^a^	3071 ± 887^b^	2486 ± 800^c^	0.006 (^a, b ^vs ^c^)

Per cycle data are presented by menotropin (hMG), highly-purified menotropin (HP-hMG) or follitropin alfa (r-hFSH). The number of cycles, indication and age-stratified data are shown (mean [± SD]).

hMG, human menopausal gonadotropin; HP-hMG, highly-purified hMG; ICSI, intracytoplasmic sperm injection; r-hFSH, recombinant human follicle-stimulating hormone; SD, standard deviation.

Gonadotropin dose adjustments were performed more often in the r-hFSH group (53.4%) than in the hMG (18.7%) and HP-hMG (20.3%) groups (p < 0.001; Table 3). The total dose of gonadotropin to achieve a pregnancy with a live birth was significantly higher in the hMG and HP-hMG compared with the r-hFSH group (p = 0.02; Table 8). The necessary dose of hMG to achieve a pregnancy was 15.7% higher than the r-hFSH dose, while the necessary dose of HP-hMG was 11% higher. These differences were still higher when considering the live birth parameter (45.3% and 19.8%, for hMG and HP-hMG, respectively) (Table 9). Because relative differences obtained from the rates cannot be statistically compared, it was not possible to calculate whether these differences were significant.

**Table 8:** Total gonadotropin dose requirement to achieve clinical pregnancy/live birth in the 865 ICSI cycles.

	**hMG**	**HP-hMG**	**r-hFSH**	**p value**
Cycles with clinical pregnancy, IU	2519 (± 684)^a^	2655 (± 612)^b^	2237 (± 582)^c^	0.002 (^a, b ^vs ^c^)
Cycles with live birth, IU	2515 (± 613)^a^	2651 (± 614)^b^	2243 (± 589)^c^	0.02 (^a, b ^vs ^c^)

Total dose of menotropin (hMG), highly-purified menotropin (HP-hMG) and follitropin alfa (r-hFSH) used per cycle resulting in a clinical pregnancy (n = 320) and per cycle resulting in a live birth (n = 251) (mean [± SD]).

hMG, human menopausal gonadotropin; HP-hMG, highly purified hMG; ICSI, intracytoplasmic sperm injection; r-hFSH, recombinant human follicle-stimulating hormone; SD, standard deviation.

**Table 9:** Mean gonadotropin doses required to achieve clinical pregnancy/live birth in the 865 ICSI cycles.

	**hMG**	**HP-hMG**	**r-hFSH**	**Relative difference, %**	
				**hMG vs r-hFSH**	**HP-hMG vs r-hFSH**
Dose per clinical pregnancy, IU*	7563	7258	6536	15.7	11
≤35 years	7202	6695	5942	21.2	12.7
>35 years	8456	8100	8020	5.4	1
Dose per live birth, IU*	10,170	8390	7000	45.3	19.8
≤35 years	9690	7739	6364	52.2	21.6
>35 years	11,371	9364	8589	32.4	9

Mean gonadotropin doses required to achieve one clinical pregnancy or one live birth are presented, with relative differences for hMG and HP-hMG compared with r-hFSH.

*Calculated as the mean dose per cycle/clinical pregnancy rate or live birth rate.

hMG, human menopausal gonadotropin; HP-hMG, highly purified hMG; ICSI, intracytoplasmic sperm injection; r-hFSH, recombinant human follicle-stimulating hormone.

## Discussion

IVF is a highly complex procedure. Before clinical pregnancy and live birth can be achieved, several critical steps are required, including the ovarian stimulation regimen. In this context, it is vital to evaluate these outcomes in routine clinical practice to account for subtle differences in patient response. As a rule, comparative, prospective and randomized clinical trials are the gold standard to evaluate the efficacy and safety of a therapy. However, some of these trials are not statistically powered to show relevant clinical differences between treatments. Moreover, strict inclusion and exclusion criteria are required, which do not represent the heterogeneous patient population typically encountered in clinical practice. Another important consideration is that many ART studies use the clinical pregnancy rate as the primary efficacy endpoint instead of live births. However, the latter may be difficult to evaluate if pregnancy outcome data are not readily available.

The current analysis examined the clinical efficacy of three different gonadotropins used for COH in ICSI cycles. In our program, clinical and laboratory data are systematically and continually entered into our patient database and managed by software specially developed for assisted reproduction programs (Androsys^®^, Brazil). Clinical follow up of pregnancies is carried out twice per month. With confirmation of live birth, follow up continues with the registration of gestational ages, birth weights, neonatal disorders, and eventual malformations for a period of 30 days post delivery. Using the Androsys^® ^database, it was possible to compare COH regimens in more than 860 consecutive ICSI cycles at a single ART center. To our knowledge, this is one of the most comprehensive comparative analyses examining both the live birth rates and the total gonadotropin dose needed per cycle resulting in clinical pregnancy and live birth. Additionally, our COH regimens consisted of the respective gonadotropin products (hMG, HP-hMG, and r-hFSH) administered as single agents, that is, combination protocols of hMG or HP-hMG plus r-hFSH were not used, a clinical approach that is widely utilized elsewhere.

Due to the retrospective, observational design of this analysis, the possibility of some inherent bias exists, although the non-selected patient population was representative of the therapeutic profile observed in current clinical practice. Outcomes were analyzed on an overall basis and stratified according to acknowledged prognostic parameters commonly used in reproductive medicine (age, infertility factor, and number of treatment attempts) to account for potential bias. For example, demographic characteristics were not homogeneous across the patient population. In fact, the mean age of the menotropin (hMG) group was significantly lower (1.5 years on average) and baseline LH values were significantly higher in the HP-hMG group than in the other groups. Moreover, more than 70% of the patients treated in the hMG group were aged ≤35 years versus approximately 60% in the other two groups. This could explain the shorter duration of stimulation observed in this group as compared with the HP-hMG and r-hFSH groups.

The three treatment groups showed similar live birth rates independent of the gonadotropin used for COH, although a trend towards higher live birth rates was observed in the r-hFSH and hMG-HP groups, in both the overall population and the subgroup aged ≤35 years. In 2004, Ludwig et al. published their analysis of more than 20,000 cycles from the IVF National Registers in Germany and found that live birth rates were 16.6% higher with r-hFSH compared with hMG when used for COH after pituitary down-regulation [10]. In their analysis, however, the authors did not evaluate the results stratified by the type of hMG (conventional or purified), although the differences favoring r-hFSH remained even after stratification for several prognostic parameters, including age. In a post-hoc analysis of a prospective, randomized trial, Platteau et al. observed that HP-hMG was associated with significantly higher live birth rates than r-hFSH when used for COH in classic IVF cycles, although they reported similar efficacy in ICSI cycles [11]. However, when evaluated in a prospective, randomized IVF trial, there was no significant difference in ongoing pregnancy rates between HP-hMG and r-hFSH [12].

Although live birth rates were similar among the three treatment groups, the spontaneous abortion rate was significantly higher in the hMG group (Table 4). However, the significant difference in spontaneous abortion rates occurred only in the subgroup of women aged >35 years. One possible explanation was the finding that the mean age was significantly higher (1.0 years on average) in the hMG subgroup of women aged > 35 years compared with the other groups (Table 5).

In Brazil, the acquisition cost of r-hFSH is about 40-50% more expensive than the urinary menotropins; until now, this was an argument that supported the choice of urinary menotropins for COH. However, in our analysis, significantly lower doses of follitropin alfa per ICSI cycle were required to achieve similar clinical pregnancy and live birth rates. This observation was valid for the overall population as well as for the subgroups stratified for age, indication, and number of treatment attempts. Moreover, significantly higher doses of both menotropin products were necessary per pregnancy and per each live birth (20-45% higher) when compared with r-hFSH.

The explanations for these findings may relate to differences in the gonadotropin products due to improved processes available through utilization of recombinant technologies [13,14]. Manufacturing improvements have resulted in significantly improved purity and specific activity, as well as batch-to-batch consistency of the follitropin alfa drug substance [14,15]. Indeed, in clinical trials, increased efficiency was observed with follitropin alfa FbM in terms of a lower total dose and shorter treatment period when compared with FSH products [16-19]. Also, as noted in the Methods, we routinely utilize a step-down ovarian stimulation protocol to avoid ovarian hyper-response. Because of the product improvements inherent with the follitropin alfa product, our clinicians are confident that dose adjustments with a decrement of 37.5 IU are clinically effective. The clinical effectiveness of 37.5 IU dosing has not been similarly demonstrated with either of the hMG products.

## Conclusion

Similar live birth rates were achieved with hMG, HP-hMG and r-hFSH when used for COH after pituitary down-regulation with GnRH-a in ICSI cycles. Significantly lower total doses of r-hFSH were administered per cycle, compared with the menotropin products. Per each live birth, considerably higher doses of hMG and HP-hMG (45.3% and 19.8%, respectively) were used for ovarian stimulation. Thus, the greater purity and consistency of the r-hFSH FbM formulation resulted in higher treatment efficiency than urine-derived gonadotropins in terms of clinical pregnancy rates and live births.

## Competing interests

Sandro C Esteves was a member of the Merck Serono International Advisory Board on Gonadotropins from February 2006 to April 2008. Joan C Schertz is an employee of EMD Serono, Rockland, MA, an affiliate of Merck KGaA, Darmstadt, Germany. Sidney Verza, Danielle T. Schneider and Silval F.C. Zabaglia have nothing to declare.

## Authors' contributions

SCE, SV, DTS and SFCZ were involved in the acquisition and analysis of the data, as well as drafting and revision of the manuscript. JCS was involved with the analysis of the data and the drafting and revision of the manuscript. All the authors read and approved the final version.
